# Genomic evidence for non-random endemic populations of decaying exons from mammalian genes

**DOI:** 10.1186/1471-2164-10-309

**Published:** 2009-07-13

**Authors:** David DeLima Morais, Paul M Harrison

**Affiliations:** 1 Department of Biology, McGill University, Stewart Biology Building, 1205 Docteur Penfield Ave, Montreal, QC, H3A 1B1, Canada

## Abstract

**Background:**

Functional diversification of genes in mammalian genomes is engendered by a number of processes, *e.g*., gene duplication and alternative splicing. Gene duplication is classically discussed as leading to *neofunctionalization *(generation of new functions), *subfunctionalization *(generation of a varied function), or *pseudogenization *(loss of the gene and its function).

**Results:**

Here, we focus on the process of pseudogenization, but specifically for individual exons from genes. It is at present unclear to what extent pseudogenization of individual exon duplications affects gene evolution, *i.e*., is it a random phenomenon, or is it associated with specific types of genes and encoded proteins, and positions in gene structures? We gathered genomic evidence for *pseudogenic exons *(ΨEs, *i.e*., exons disabled by frameshifts and premature stop codons), to examine for significant trends in their distribution across four mammalian genomes (specifically human, cow, mouse and rat). Across these four genomes, we observed a consistent population of ΨEs, associated with 0.4–1.0% of genes. These ΨE populations exhibit codon substitution patterns that are typical of an endemic population of decaying sequences. In human, ΨEs have significant over-representation for functional categories related to 'ion binding' and 'nucleic-acid binding', compared to duplicated exons in general. Also, ΨEs tend to be associated with some protein domains that are abundant generally, *e.g*., Zinc-finger and immunoglobulin protein domains, but not others, *e.g*., EGF-like domains. Positionally, ΨEs are also significantly associated with the 5' end of genes, but despite this, individual stop codons are positioned so that there is significant avoidance of potential targeting to nonsense-mediated decay. In human, ΨEs are often associated with alternative splicing (in 22 out of 284 genes with ΨEs in their milieu), and can have different parts of their sequence differentially spliced in alternative transcripts. Some unusual cases of ΨEs embedded within 5' and 3' non-coding exons are observed.

**Conclusion:**

Our results indicate the types of genes that harbour ΨEs, and demonstrate that ΨEs have non-random distribution within gene structures. These ΨEs may function in gene regulation through generation of transcribed pseudogenes, or regulatory alternate transcripts.

## Background

Natural selection acts on phenotypes arising from a vast range of genomic variations: chromosomal and segmental duplications, local duplications, and smaller insertions, deletions and nucleotide substitutions. Local duplication arises not only for whole genes or multiples of genes, but also for pieces of genes and for individual exons.

A pseudogene (ΨG), in the case of protein-coding genes, is a copy of a gene that has symptoms of protein-coding deficiency [1-6]. Symptoms of protein-coding deficiency include: *(i) *coding-sequence disablements (frame-shifts and premature stop codons); *(ii) *neutral codon substitution patterns (that yield values of K_a_/K_s_, the ratio of non-synonymous to synonymous codon substitutions of ~1.0); *(iii) *protein domain truncations [2]; *(iv) *mutation of deeply-conserved residue positions essential for protein function or structural integrity [1]. Processed pseudogenes are made by reverse transcription and re-integration into the genome, and have been extensively studied elsewhere [1-6]. Non-processed pseudogenes can arise after local or segmental gene duplication, and subsequent loss of protein-coding ability through mutation. A similar situation can arise within an individual gene structure: one or more exons can become duplicated within the vicinity of a gene. Such partial gene duplications may then lose coding ability, becoming *pseudogenic exons *(ΨEs), in a similar way.

Here, we have gathered genomic evidence for the distribution of *pseudogenic exons *(ΨEs) in the chromosomal milieu of annotated genes of four mammals with high-coverage genome assemblies and extensive transcriptional validation (human, cow, mouse and rat). Such ΨEs can have a functional role. For example, recently it has been described that ΨEs with stop codons that are alternatively spliced can target messenger RNAs to nonsense-mediated decay (NMD), in a way that causes changes in expression levels for other transcripts from the gene [7]. In our analysis, we define ΨEs specifically using coding-sequence disruptions (*i.e*., frameshifts and premature stop codons). We find a non-random distribution of ΨEs in each mammalian genome, associated with certain subtypes of genes and positions within genes.

## Results and discussion

**Figure 1:**
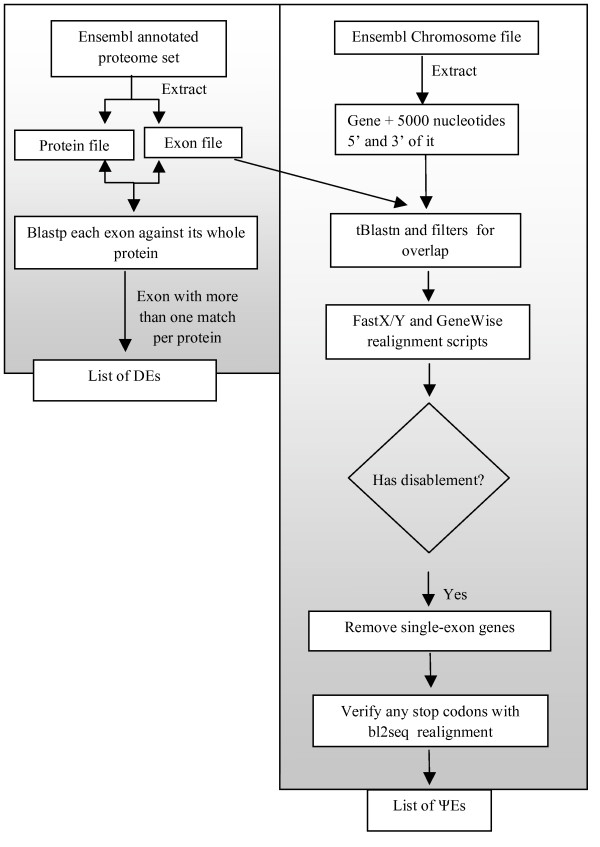
**Pipeline annotation of DEs and ΨEs**. The pipeline annotation is summarized.

A pipeline was derived to detect *pseudogenic exons *(ΨEs) in the immediate chromosomal *milieu *of genes (Figure 1; see *Methods *for details). A ΨE is defined as an exon copy whose coding ability is compromised by a frameshift or a premature stop codon. Such frameshifts and stop codons are the most obvious indicators of coding-sequence decay. The designated *parent exon *for a ΨE is the most similar exon in the surrounding annotated gene structure. In addition, we annotated duplicated exons (DEs) in the transcripts from each gene, as described in *Methods*.

We focused on four mammalian genome assemblies with high (>7X) coverage (human, cow, mouse and rat), to analyze the extent of the occurrence of ΨEs. We examined for significant trends in the distribution of ΨEs for a variety of properties. In particular, we focussed on assessing the peculiarities of the ΨEs in comparison to the general population of duplicated exons. We analyzed the following: *(i) *divergence from designated parent exons; *(ii) *association with protein families; *(iii) *association with Gene Ontology functional categories; *(iv) *position of ΨEs with respect to the intron-exon structure of the gene; *(v) *participation in alternative splicing, and *(vi) *coding-sequence selection pressures, as judged by K_a_/K_s _values.

Table 1 summarizes the distribution of ΨEs. Strikingly, ΨEs occur at a consistent level across all of the mammalian genomes studied. The annotation pipeline identified between ~300 to ~600 cases of ΨEs per genome. These ΨEs occur for 0.4–1.0% of genes, with a frequency of 1.3–2.0 ΨEs per gene. In addition, we determined ~4000–7000 duplicated exons (DEs) within the annotated genes of each of the four studied mammals (Table 1). A substantial fraction (~12–22%) of the ΨEs are located on the strand opposite to the putative parent gene (Table 1), indicating some sort of inversion process in their generation.

**Table 1:** Summary of the annotations

Feature	*Homo sapiens†*	*Mus musculus†*	*Rattus norvegicus†*	*Bos taurus†*
**DE**	6717 (1341)	4645 (1079)	4052 (993)	4389 (982)
**ΨE**	377 (284)	270 (209)	364 (218)	581 (298)
- 5' half	263	178	88	431
- 3' half	114	92	276	155
- Opposite strand	13%	12.2%	21.5%	14.31%
Number of **ΨEs **that would lead to NMD targeting	55	48	138	194
- Orthologs and the Gene Order test	-------	36/67* (53.7%)	39/62* (62.9%)	45/75* (60%)

* Number that can be assigned orthologs, as determined in Ensembl annotation. We can see that most assigned orthologs are in syntenic positions, with the exception of the dog genome.

† The number of genes bearing the exons is in brackets.

### (i) Divergence from designated parent exons

**Figure 2:**
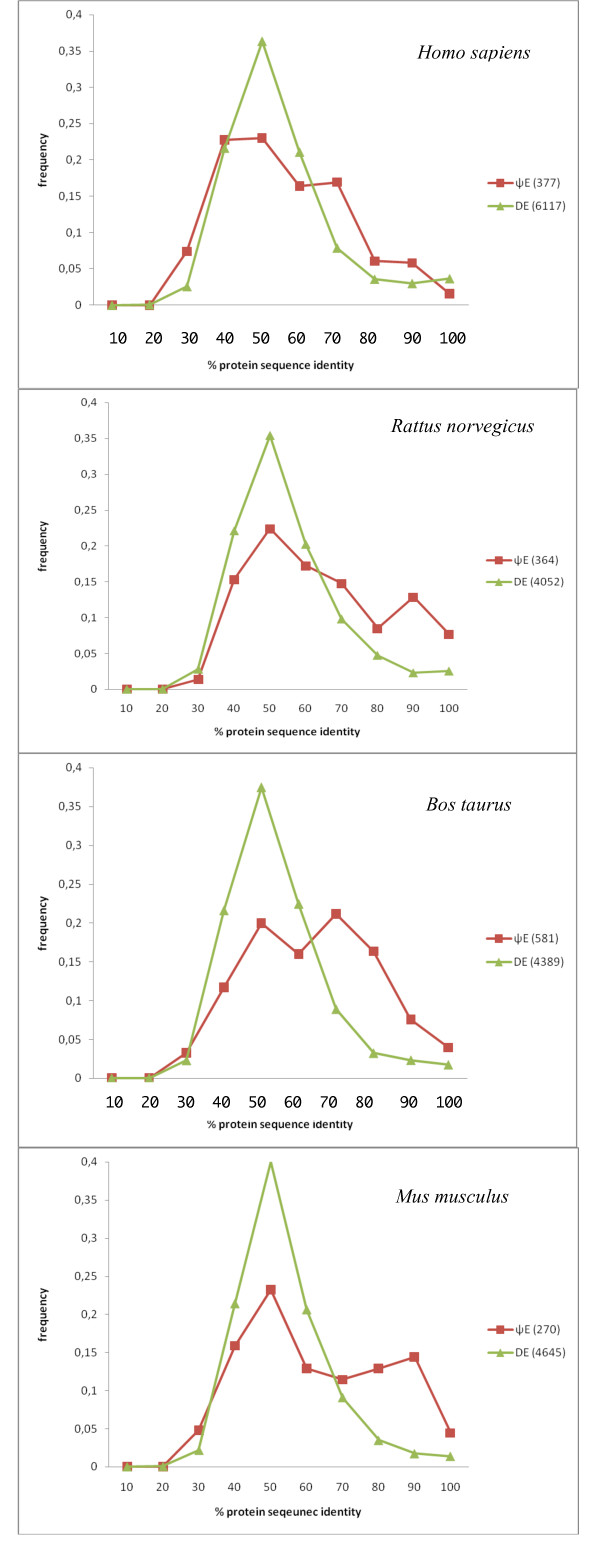
**Distributions of protein sequence identity for DEs and ΨEs**. These curves are for the data sets listed in Table 1. There are four panels for each of the four mammals analysed, labelled with the binomial species name. For each panel, the DE curve is green, and the ΨE curve is red. The bin label *x *is for all values such that, *x*-10 < value ≤ *x*.

We analyzed the distribution of percentage sequence identity between the ΨEs and their respective designated parent exons. These distributions were compared to an equivalent distribution for DEs (Figure 2). This equivalent distribution is from comparison of the DEs to their most homologous exons within the same gene. The distributions generally have a mode for both DEs and ΨEs at 40–50% (Figure 2). Therefore, ΨEs are not unusually divergent in terms of protein sequence identity with respect to DEs in general.

**Figure 3:**
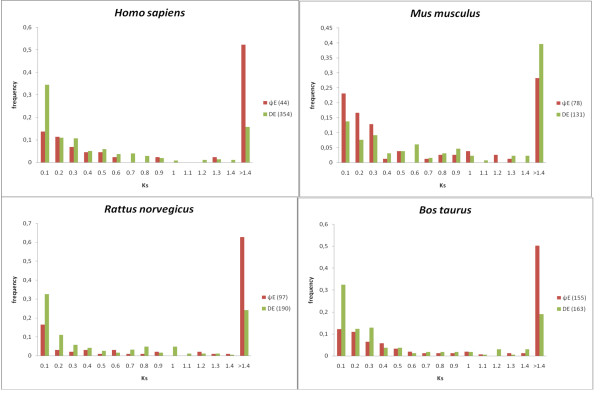
**Distributions of Ks for DEs and ΨEs**. These curves are for the data sets listed in Table 1. The DE curve is green, and the ΨE curve is red. The bin label *x *is for all values such that, *x*-0.1 < value ≤ *x*.

In addition, we examined distributions of K_s _values for those exons which align to their designated parent exons with > = 70% amino-acid sequence identity (to avoid consideration of sequences with codon saturation) (Figure 3). Although recently, evidence has been uncovered indicating that K_s _values are under selection in mammals [8], they can still be used in a comparative sense to compare the age trends in populations of sequences. In general, there is a notable tendency for very young exon duplications, with a peak appearing in the K_s _distributions for all species at the interval 0.00–0.10, for ΨEs and for duplicated exons in general. Interestingly, also, a sizeable fraction of ΨEs appear to be derived from anciently duplicated exons (i.e., 30–60% having K_s _> 1.4); such exons were likely duplicated earlier in vertebrate evolution, and became disabled later during mammalian speciation.

**Figure 4:**
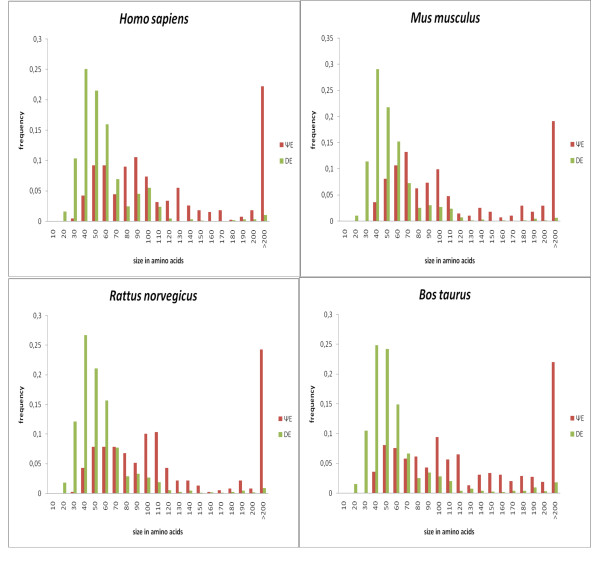
**Distributions of size (in nucleotides) for DEs and ΨEs**. These curves are for the data sets listed in Table 1. The DE curve is green, and the ΨE curve is red. The bin label *x *is for all values such that, *x*-10 < value ≤ *x*.

**Figure 5:**
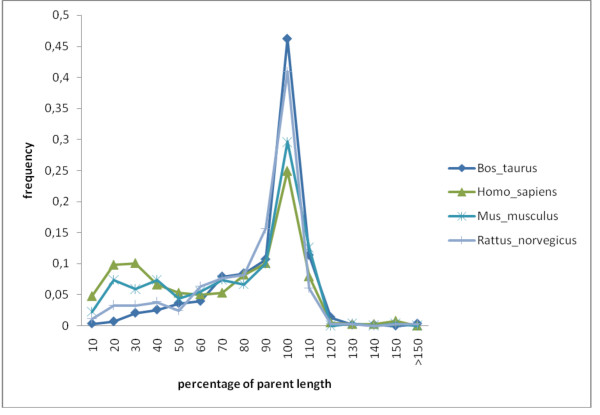
**Distributions of fraction of length of parent exon for ΨEs**. The bin label *x *is for all values such that, *x*-10.0 < value = *x*.

The distribution of exon sizes of DEs has medians in the range ~40–50 amino acid residues (Figure 4, Additional File 1). However, ΨEs are substantially longer than DEs in general (median values in the range 70–110 amino acid residues, and broader distributions) (Figure 4). This larger size trend for ΨEs arises chiefly from the exon size trends for the specific gene families that tend to make large numbers of ΨEs, such as the Zinc-finger-containing (ZFC) genes (see Additional File 2 and protein family section below). In aggregate, the majority of the ΨEs (> ~75%) have at least half of their designated parents' length, and ~55% have between 0.9–1.1 of their parents' length (Figure 5). A small percentage (6–13%) of the ΨEs are marginally longer than their parent exons (Figure 5); this is potentially because of neutrally-occurring insertions arising after duplication [9].

### (ii) Association with protein families

Some gene families spawn large numbers of pseudogenes. Examples include olfactory receptors [10], ribosomal-protein genes [11], ABC transporters [12], and heat shock proteins [13]. We noted previously that the gene families with the most non-processed pseudogenes tend to be involved in some form of interaction with the environment [1], *e.g*. through roles in immunity [14], chemosensation [1,15], or small-molecule transport [12]. Such gene families can also be linked to recent segmental duplications in mammals [16]. Here, we examined which are the most common protein domain families in the ΨE and DE data sets (Additional File 2). These numbers indicate the number of exons with at least one copy of each protein domain considered. Exons containing zinc-finger domains and immunoglobulin-like domains are consistently in the top five most abundant for both ΨEs and DEs. Genes for zinc-finger-containing (ZFC) proteins have undergone lineage-specific expansions over the course of mammalian evolution, so decaying ZFC exons are an expected consequence of this, and could perform regulatory roles as part of transcribed pseudogenes [17]. Transcribed pseudogenes have recently been shown to regulate the expression of homologous genes through the formation of small, interfering RNAs [18,19]. Immunoglobulin-like domains are used in many proteins that are involved in various aspects of immunity, and have been previously noted to generate large numbers of pseudogenes [14]. The most notable difference between ΨEs and DEs in general, is that ΨEs rarely arise that contain EGF-like (epidermal growth factor-like) domains, whereas these exons are consistently abundant, generally (significant difference, P < 0.05, binomial statistics; Additional File 2). EGF-like domains have expanded greatly in number over the course of mammalian evolution, and are found (with a small number of exceptions) either in the extracellular part of transmembrane proteins or in secreted proteins [20,21].

### (iii) Association with Gene Ontology functional categories

We used Gene Ontology (GO) functional classification to assess which functional associations are the most common for ΨEs (Table 2). A pairwise comparison between lists of genes was performed to check over-represented terms according to various criteria, for ΨEs, and for DEs generally. In this analysis, we only studied the human, mouse and rat genomes, since these are the genomes with extensive GO functional annotation. Specifically of interest are the GO terms that are over-represented in ΨEs compared to DEs (Table 2). Significant over-representation is calculated using a Fisher's exact test with P' < 0.05, and a correction to P' for multiple hypothesis testing [22].

**Table 2:** Most common Gene Ontology functional categories †

*Homo sapiens*		
All genes (Total = 31524)	ΨEs (Total = 284)	DEs (Total = 1341)

GO:0005515, protein binding (5864)	GO:0043167, ion binding (92)*^§^	GO:0005515, protein binding (372)
GO:0043167, ion binding (3861)	GO:0003676, nucleic acid binding (74)*^§^	GO:0043167, ion binding (349)*
GO:0003676, nucleic acid binding (3251)	GO:0005515, protein binding (34)	GO:0003676, nucleic acid binding (176)
GO:0016787, hydrolase activity (2053)	GO:0016740, transferase activity (18)	GO:0016787, hydrolase activity (105)
GO:0000166, nucleotide binding (1992)	GO:0004872, receptor activity (13)	GO:0004872, receptor activity (84)
GO:0004872, receptor activity (1765)	GO:0000166, nucleotide binding (12)	GO:0000166, nucleotide binding (57)
GO:0016740, transferase activity (1631)	GO:0016491, oxidoreductase activity (11)^§^	GO:0016740, transferase activity (36)
GO:0016491, oxidoreductase activity (723)	GO:0016787, hydrolase activity (10)	GO:0030246, carbohydrate binding (35)*
GO:0015075, ion transporter activity (541)	GO:0030246, carbohydrate binding (6)	GO:0005201, extracellular matrix structural constituent (22)*
GO:0008289, lipid binding (420)	GO:0046906, tetrapyrrole binding (3)	GO:0004857, enzyme inhibitor activity (21)

*Mus musculus*		

All genes (Total = 28390)	ΨEs (Total = 209)	DEs (Total = 1079)

GO:0005515, protein binding (5553)	GO:0043167, ion binding (55)*	GO:0005515, protein binding (374)*
GO:0043167, ion binding (3672)	GO:0003676, nucleic acid binding (43)*	GO:0043167, ion binding (321)*
GO:0003676, nucleic acid binding (3382)	GO:0004872, receptor activity (27)^§^	GO:0003676, nucleic acid binding (173)
GO:0004872, receptor activity (2779)	GO:0016787, hydrolase activity (16)	GO:0016787, hydrolase activity (114)
GO:0016787, hydrolase activity (2260)	GO:0005515, protein binding (14)	GO:0004872, extracellular matrix (109)
GO:0000166, nucleotide binding (2061)	GO:0016491, oxidoreductase activity (9)	GO:0000166, nucleotide binding receptor activity (91)
GO:0016740, transferase activity (1805)	GO:0004857, enzyme inhibitor activity (7)	
GO:0016491, oxidoreductase activity (911)	GO:0000166, nucleotide binding (7)	GO:0016740, transferase activity (51)
GO:0015075, ion transporter activity (598)	GO:0046906, tetrapyrrole binding (6)	GO:0030246, carbohydrate binding (41)*
GO:0008289, lipid binding (401)	GO:0016740, transferase activity (5)	GO:0005201, structural constituent (40)*
		GO:0016491, oxidoreductase activity (25)

*Rattus norvegicus*		

All genes (Total = 27302)	ΨEs (Total = 218)	DEs (Total = 993)

GO:0005515, protein binding (2732)	GO:0043167, ion binding (23)	GO:0043167, ion binding (158)*
GO:0043167, ion binding (2238)	GO:0016740, transferase activity (16)^§^	GO:0005515, protein binding (155)*
GO:0004872, receptor activity (2063)	GO:0003676, nucleic acid binding (15)	GO:0003676, nucleic acid binding (74)
GO:0003676, nucleic acid binding (1720)	GO:0004872, receptor activity (14)	GO:0016787, hydrolase activity (62)
GO:0000166, nucleotide binding (1406)	GO:0005515, protein binding (12)	GO:0000166, nucleotide binding (46)
GO:0016787, hydrolase activity (1331)	GO:0016787, hydrolase activity (12)	GO:0004872, receptor activity (37)
GO:0016740, transferase activity (1179)	GO:0000166, nucleotide binding (10)	GO:0016740, transferase activity (29)
GO:0016491, oxidoreductase activity (594)	GO:0016491, oxidoreductase activity (6)	GO:0016491, oxidoreductase activity (16)
GO:0015075, ion transporter activity (392)	GO:0046906, tetrapyrrole binding (5)	GO:0030246, carbohydrate binding (16)
GO:0003735, structural constituent of ribosome (284)	GO:0030246, carbohydrate binding (4)	GO:0005201, extracellular matrix structural constituent (14)*

* Over-represented term when compared with all genes.

§ Over-represented term when compared with DEs.

† GO term counts are listed for human, mouse and rat.

The top ten human DEs and ΨEs GO terms do not differ greatly from each other, in each of the species studied. However, each organism has distinct significant over-representations of GO terms. In the human genome, '*Ion binding' *and '*Nucleic acid binding' *are significantly over-represented in ΨEs, compared to DEs (Table 2). This overrepresentation appears to be chiefly due to ZFC transcription factors, which are obviously candidates for regulation through unproductive splicing and translation, or through the formation of regulatory transcribed pseudogenes. In mouse, 'receptor activity' is significantly over-represented in ΨEs compared to DEs, and 'transferase activity' in rat. These indicate that different types of gene have undergone pseudogenic exon formation in recent evolutionary time in each of these three organisms.

### (iv) Position of ΨEs with respect to the intron-exon structure of the annotated gene

In general, the majority of ΨEs are located within the 5' half of the genes in every studied genome (P < 0.01, using χ^2 ^tests; Table 1). This scenario suggests that proteins tend to become more complex through addition of exons to the 5' termini of their encoding genes. These exons could be inefficiently spliced and therefore will appear in only a few transcripts, while they may be selected against if they disrupt the normal gene function [23,24]. Interestingly, the ΨEs are significantly 5' of their parents in rat (Table 3). We suggest that this is due to lineage-specific activity related to specific gene families (Additional File 2).

**Table 3:** Position of ΨEs in related with their parents

	Number of ΨE 5' to parent	Number of ΨE beyond the 5' end of the gene	Number of ΨE 3' to parent	Number of ΨE beyond the 3' end of the gene
*Homo sapiens*	179	78	198	118
*Mus musculus*	131	54	139	84
*Rattus norvegicus*	213 †	42	151 †	51
*Bos taurus*	298	34	283	41

† Significantly non-random, P < 0.05, chi-squared test.

A key issue in examining the distribution of stop codons in ΨEs, is whether they would produce transcripts that are susceptible to nonsense-mediated decay (NMD). We examined for individual stop codons in the ΨEs that would lead to NMD targeting (Table 1). The number of such stop codons in ΨEs that would lead to NMD is significantly smaller than what is expected by chance (P < 0.01, using χ^2 ^test), in human and cow, but not in the two rodent genomes. The expected distribution in this case, is calculated from the total size of the gene introns divided appropriately, given the position of the stop codons in each ΨE. This indicates a selection pressure in human and cow, against the positioning of individual stop codons in ΨEs in places that would cause NMD. It has been shown that alternative splicing can be coupled to NMD to regulate the expression of other transcripts from a gene [25]. This mechanism has been dubbed *regulated unproductive splicing and translation *[25]. There may therefore be a selection pressure against placement of stop-codon-bearing exons in some genes, so that they are not affected by this mechanism.

We curated on the human ΨE data, to search for unexpected positional distributions in genes. In human, forty-five ΨEs were found embedded in an untranslated region (UTR). These UTR-embedded ΨEs are not highly conserved. Only eight of them are also found in chimp and rhesus (four in each species), and none of them are shared by the three primate species simultaneously. None of the embedded ΨEs is conserved in a non-primate species (cow, dog, mouse or rat). This is despite syntenic conservation of 28 out of the 45 genes in a non-primate species involved in the embedding, when manually compared in the UCSC Genome browser [26]. It is possible that these UTR-embedded ΨEs are remnants of overlapping gene arrangements. The manner of overlap for overlapping gene pairs changes very dynamically over evolution; for example, only 95 out of 255 human overlapping gene pairs were reported to be conserved as overlapping pairs in the mouse genome [27].

### (v) Participation in alternative splicing

Alternative splice products containing premature stop codons can be degraded through nonsense-mediated decay (NMD), and consequently cause altered expression of protein-coding transcripts through changes in abundance of splicing factors [7]. We examined whether any ΨEs have been annotated as part of alternative splicings. To do this, we cross-referenced the ASD alternative splicing database [28] 'splicing event' annotation, with our ΨE list from the human genome. Of the human 284 genes that harbour a ΨE in their genomic milieu, 101 are present in the ASD alternative splicing database. Out of these, we found 22 genes (entailing 59 transcripts) with evidence of transcription of a ΨE as an alternative exon. Analyzing the alternatively-spliced forms in detail, we found four cases of an unusual topology of splicing (Additional File 3). These four human ΨEs can be differentially spliced in a topologically novel manner, in which one portion of a ΨE is recruited in one splice form, while a different portion of it can take part in another splice form (Additional File 3).

### (vi) K_a_/K_s _analysis

**Figure 6:**
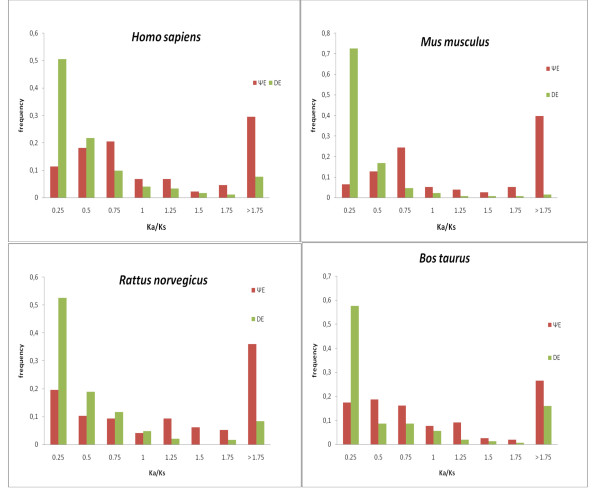
**Histograms of K_a_/K_s _for for DEs and ΨEs**. The DE histogram is green, and the ΨE histogram is red. The bin label *x *is for all values such that, *x*-0.25 < value ≤ *x*.

K_a_/K_s _(*i.e*., the normalized ratio of non-synonymous and synonymous codon site substitution rates) is a measure of selection on coding sequences; values < 1.0 can indicate purifying selection, whereas values ~1.0 are theoretically expected for neutral selection pressures. Values significantly > 1.0 indicate positive selection over the whole of a sequence. We examined K_a_/K_s _values for the different populations of ΨEs and DEs. K_a_/K_s _values were calculated for all exon alignments with amino-acid sequence identity > 70%, to avoid consideration of saturated nucleotide sequences [2,3]. In general, the DEs exhibit a mode in the range 0.00–0.25 for K_a_/K_s_, indicating a tendency to purifying selection (Figure 6). In contrast, the ΨE populations do not exhibit such a mode, instead peaking in the range 0.25–0.75 (Figure 6). We have previously observed such a K_a_/K_s _peak for pseudogenic transcripts captured by transposons [29], and for processed pseudogenes [3]. Thus is to be expected for endemic populations of neutrally evolving sequences, from comparisons with their putative parent sequences. The reasons for such K_a_/K_s _values < 1.0 may include: *(i) *continued purifying selection on the putative parent sequence; *(ii) *an original protein-coding phase for the present-day ΨE. Interestingly, ~30% of ΨE cases, have K_a_/K_s _values > 1.5, which indicates that they may have undergone positive selection before becoming disabled.

## Conclusion

We gathered genomic evidence to assess for non-random distribution of *pseudogenic exons *(ΨEs) in four mammalian genomes. We observed endemic populations of decaying exons consistently across genomes, arising for up to ~1% of genes. These ΨEs were defined using coding-sequence disablements (frameshifts and premature stop codons). Of course, other pseudogenic exons may exist (such as those arising from initial disablement of splicing signals); however, such pseudogenic exons would be likely to acquire coding-sequence disablements rapidly, soon after their initial disablement.

The *pseudogenic exons *(ΨEs) are longer than duplicated exons in general, are associated with genes encoding specific protein domain families, such as zinc-finger-containing proteins, and are noticeably lacking for genes containing domains that are otherwise abundant, such as EGF-like domains. The ΨEs also demonstrate species-specific over-representation of GO functional categories relative to duplicated exons in general; for example, in human, GO functional categories for 'ion-binding' and 'nucleic acid binding' are significantly over-represented, compared to duplicated exons generally. The ΨE populations indicate the sorts of genes that have undergone exon decay in recent mammalian evolution (recent enough, and in large enough amounts, for them not to be deleted from the genomic DNA). We find statistical evidence for selection pressure on avoidance of stop codon placements in ΨEs that would lead to nonsense-mediate decay. In addition, we find some interesting positionings of ΨEs in gene structures, such as embedding in UTRs, or partial alternative splicing. The ΨE populations are a potential resource for the formation of transcribed pseudogenes, which can function in the regulation of homologous genes through formation of small, interfering RNAs [18,19,30]. They may also be involved in alternative transcripts that have a regulatory function [7]. The annotated ΨEs that we have analysed will be a fertile source for study using large-scale micro-array expression techniques for these two potential regulatory functions. Also, the ΨE data sets will be useful for further gene evolution study in mammals. The data are available from the authors at .

## Methods

### Genome data

The genome sequences and annotation files of four mammals analyzed in this paper (human, mouse, rat, and cow) were downloaded from the Ensemble Web site , in February 2007. The genome assemblies are: human = Homo_sapiens.NCBI36.43; mouse = Mus_musculus.NCBIM36.43; rat = Rattus_norvegicus.RGSC3.4.43; cow = Bos_taurus.Btau_3.1.43. These genomes were chosen, because: *(i) *the genome assemblies are to high (>7X) coverage, and *(ii) *>85% of the gene annotations in these genomes have complete transcription validation. To identify the duplicated exons we compared each exon of each gene against the whole protein sequence of the same gene using BLASTp (e-value ≤ 10^-4^) [31]. Exon definitions were taken directly from the genome annotations. To detect ΨEs, each exon was compared with the whole genomic DNA of the same gene plus a 5000-nucleotides (nt) buffer, 5' and 3' of the gene (Figure 1). The vast majority (>85%) of the introns of mammalian protein-coding genes are <5000 nt in length. As is illustrated with the data from the cow genome in Additional File 4, the number of ΨEs that are detected, has only a small dependence on the size of this buffer. We used protein-level sequence alignment to detect ΨEs throughout the paper; this is so that we can exploit the signal of protein coding sequence that is still in these sequences to detect them in the genomic DNA.

### Identification of Duplicated Exons (DEs) and Pseudogenic Exons (ΨEs)

#### (1) Exon boundaries

The positioning of exon boundaries in encoded protein sequence was deduced and extracted from Ensembl Genbank-style annotation files, downloaded from . The positioning was then used to map the exact location of an exon BLAST match [31].

#### (2) Homology detection

Each exon (amino acid sequence) was compared against its whole protein sequence using BLASTp to find duplicated exons with similarities with e-value ≤ 10^-4^. For ΨEs, each exon was compared (using tBLASTn [31], with e-value as above) against the genomic milieu of the encoding gene, which is defined as the genomic DNA of the gene (including introns), plus 5000 nucleotides, 5' and 3' of the gene.

#### (3) Fastx/y and Genewise realignments

After filtering for overlapping each tBLASTn match was realigned using FASTX/Y, as previously described [2,15,32]. The FASTX/Y [33] program allows longer alignments and also allows the identification of stop codon and frame-shifts in ΨEs. To confirm that the disablements of FASTX/Y were not an artifact we also aligned the ΨEs with GeneWise [34]. Only ΨEs confirmed by both methods were kept in our analyses.

#### (4) Filtering

ΨEs were filtered to remove olfactory receptors (ORs) and other single exon genes, since it is difficult to classify them as processed or duplicated pseudogenic exons [35]. Each match was compared with the Interpro , Gene Ontology (GO [36]) descriptions and Ensembl  protein family descriptions. If a ΨE was annotated in at least one of those databanks as an OR or other single-exon gene, it was removed from the analysis. To confirm the presence of stop-codons each ΨEs was realigned against its translated parent using bl2seq [37]; the output was parsed so that stop codons outside of a margin of 10 amino acids at the ends of the aligned subsequences were adjudged to be verified.

#### (5) Orthologs

The information about orthologs was extracted from the Biomart query system in the Ensembl database. As a further filter for the ortholog assignments, we performed a 'local gene order' test [38]. We compared the chromosomal milieu of genes bearing ΨEs, with the milieus of their orthologs, as follows. After identifying the ortholog of the gene containing the ΨE (step (5) above), we took a window (W_genes_) of 9 genes in either direction (the gene bearing the ΨE plus 4 genes 5' and 4 genes 3' of it) and 'BLASTed' against the equivalent 9 genes for the ortholog. We focused on the human genome; therefore, this local gene order test was performed for the human data vs. cow, dog, chimp, rhesus, mouse and rat genomes. The number of significant matches (BLASTp e-value ≤ 10^-4^, sequence identity >45%, and match ≥ 0.6 length of both orthologs) between the milieu of the two considered species was investigated. We allowed up to three gaps in total within the W_genes _windows.

#### (6) NMD targeting

To analyse for potential NMD targeting, we disregarded any ΨE located beyond the 5' and 3' UTRs, and also ΨEs located after the real stop codon, since they would not lead to nonsense-mediated decay (NMD). Then, we mapped the position of each stop codon in every ΨE to see if they are in a NMD area. If a stop codon would be located more than 55 nucleotide 5' to the last exon-exon junction in a transcript containing the ΨE, then the ΨE was labeled as within a putative NMD target.

### Functional categories

Gene Ontology (GO [36]) functional categories, Ensembl protein family and pfam protein family descriptions, where retrieved using the Biomart tool [39]. GO functional category enrichment analyses of DEs and ΨEs were performed using FatiGo database [22].

### Alternative splicing (AS)

We checked whether exons are alternatively spliced, by counting up the number of splice forms that a gene produces, and labeling the exons as *constitutive *if they appear in all splice forms, and *alternative *otherwise. We cross-referenced the coordinates of each ΨE with every event of alternative splicing annotation in the ASD database database [28].

### Analysis of Ka/Ks values

The program codeml of the PAML package [40] was used to calculate the maximum-likelihood Ka/Ks values of designated parent exons compared to ΨEs and DEs. The input codon alignments were generated using the PAL2NAL program [41]. Only pairs of sequence with ≥ 70% of identity and ≥ 40 amino acids long were used in this analysis as the reliability of Ka/Ks analysis falls rapidly below this threshold [42].

## Authors' contributions

DM performed the data analysis and wrote the initial draft of the manuscript. PH conceived and directed the project, and wrote later drafts of the manuscript. All authors read and approved the final manuscript.

## Supplementary Material

Additional file 1 **Statistics of exon length**. Statistics of exon length in human, mouse, rat and cow. Click here for file

Additional file 2 **Table showing the most abundant protein domains for exon types**. Table showing the most abundant protein domains for exon types. Click here for file

Additional file 3 **Table of splice forms**. Cases of alternative recruitment from different parts of apparently pseudogenic exons. Click here for file

Additional file 4 **Examination of buffer dependence**. Number of ΨEs detected, as a function of 5'/3' buffer size in the cow genome. Click here for file
