# Supplementary material for: Genomic evidence for non-random endemic populations of decaying exons from mammalian genes
Source: BMC Genomics. 2009 Jul 13;10:309. doi: 10.1186/1471-2164-10-309 (PMC2718932; doi:10.1186/1471-2164-10-309)
Supplement: Additional file 2 — Table showing the most abundant protein domains for exon types. Table showing the most abundant protein domains for exon types. [file 1471-2164-10-309-S2.doc]

|  | **ΨE†** | | | | **DE†** | | | |
| --- | --- | --- | --- | --- | --- | --- | --- | --- |
|  | **Pfam ID** | **Family name** | **% of total occurrences** | **Number of occurrences** | **Pfam ID** | **Family name** | **% of total occurrences** | **Number of occurrences** |
| *Bos taurus* | PF00096  PF01352  PF07686  PF00047  PF07679 | zf-C2H2  KRAB  V-setT  Ig  I-set | 12.5  9.7  5.9  5.2  2.0 | 36  28  17  15  6 | PF00008  PF00096  PF00047  PF07686  PF07645 | EGF  zf-C2H2  Ig  V-setT  EGF_CA | 4.9  3.7  3.2  2.9  2.5 | 85  64  55  50  43 |
| *Homo sapiens* | PF00096  PF01352  PF07686  PF00047  PF00201 | zf-C2H2  KRAB  V-setT  Ig  UDPGT | 16.6  11.3  9.7  9.4  2.5 | 72  49  42  41  11 | PF00008  PF00096  PF00047  PF07686  PF00041 | EGF  zf-C2H2  Ig  V-setT  fn3 | 4.4  3.5  3.4  3.1  2.9 | 120  97  93  85  79 |
| Mus musculus | PF00096  PF01352  PF00047  PF07686  PF00001 | zf-C2H2  KRAB  Ig  V-setT  7tm_1 | 14.1  10.5  7.6  7.6  2.9 | 39  29  21  21  8 | PF00008  PF00047  PF00096  PF07686  PF07679 | EGF  Ig  zf-C2H2  V-setT  I-set | 4.5  3.6  3.0  3.0  2.7 | 107  85  72  71  65 |
| *Rattus norvegicus* | PF00047  PF07686  PF00096  PF01352  PF04822 | Ig  V-setT  zf-C2H2  KRAB  DUF622 | 10.5  10.0  5.4  3.8  3.8 | 39  37  20  14  14 | PF00008  PF00047  PF00096  PF07686  PF07645 | EGF  Ig  zf-C2H2  V-setT  EGF_CA | 4.5  3.7  3.7  3.6  2.6 | 99  81  81  79  57 |
|  |  |  |  |  |  |  |  |  |

† The domains are counted up on a 'one per sequence' basis.
