# Supplementary material for: Genomic evidence for non-random endemic populations of decaying exons from mammalian genes
Source: BMC Genomics. 2009 Jul 13;10:309. doi: 10.1186/1471-2164-10-309 (PMC2718932; doi:10.1186/1471-2164-10-309)
Supplement: Additional file 3 — Table of splice forms. Cases of alternative recruitment from different parts of apparently pseudogenic exons. [file 1471-2164-10-309-S3.doc]

| Ensembl Gene | Ensembl Transcript | # of Splice forms | Coordinates (genomic) ***** | ΨE coordinates | EST name |
| --- | --- | --- | --- | --- | --- |
| ENSG00000174652 | ENST00000361451 | 1 | **9384637..9384722**, 9385595..9385755, 9386044..9386162, 9399612..9399765, 9400531..9400779, 9400935..9401068, 9401199..9401325, 9401922..9402002, 9404088..9404176, 9405110..9407226 | 9384640..9384968 | AY376240 |
|  |  | 4 | **9384680..9384722**, 9384971..9385032, 9385595..9385755, 9386047..9386162, 9399612..9399765, 9400541..9400652 | 9384640..9384968 | CN353236 |
| ENSG00000167766 | ENST00000301096 | 1 | **57807473..57807516**, 57810675..57810762, 57826766..57826892, 57831031..57831301 | 57807379..57808235 | DA162889  DA592481  DA347859 |
|  |  | 3 | **57807427..57807516**, 57810675..57810762, 57831031..57831343 | 57807379..57808235 | DA762987 |
|  |  |  | 57807443..57807516, 57810624..57810762, 57831031..57831542 | 57807379..57808235 | DA731895  AK027518 |
| ENSG00000094631 | ENST00000376619 | 1 | 48545431..48545493, 48546014..48546136, 48546222..48546350, 48546479..48546567, 48548789..48548873, 48548982..48549022, 48549719..48549815, 48549960..48550057, 48551384..48551488, 48551609..48551677, 48557791..48557917, 48558026..48558091, **48558185..48558244**, 48558313..48558402, 48558735..48558841, 48558926..48558993, 48559235..48559403, 48559492..48559620, 48559816..48559984, 48560677..48560810, 48561392..48561460, 48561571..48561763, 48563457..48563606, 48565974..48566148, 48566266..48566942, 48567026..48567139, 48567276..48567422, 48567520..48567648, 48567898..48568324 | 48558186..48558403 | AJ011972  AB020708 |
| ENSG00000094631 | ENST00000376619 | 4 | 48557859..48557917, **48558026..48558244,** 48558313..48558841, 48558926..48558993, 48559235..48559403, 48559492..48559620, 48559816..48559984, 48560677..48560810, 48561392..48561460, 48561571..48561763, 48563457..48563606, 48565974..48566148, 48566266..48566942, 48567026..48567139, 48567276..48567422, 48567520..48567648, 48567898..48568324 | 48558186..48558403 | AK122954 |
|  |  | 8 | 48558116..48558402, 48558735..48558841, 48558926..48558993, 48559235..48559403, 48559492..48559527 | 48558186..48558403 | BX644567 |
| ENSG00000158691 | ENST00000361028 | 1 | 28454716..28454788, **28455972..28456441**, 28461399..28461543, 28462703..28462859 | 28455962..28456525 | BQ229288 |
|  |  | 4 | **28455969..28456441**, 28461365..28461543, 28462703..28462873 | 28455962..28456525 | BQ921129 |
|  |  | 6 | 28454738..28454788, **28455972..28456477** | 28455962..28456525 | CN307911 |

* In bold are the parts of the ΨEs retained by particular transcripts.
