# Supplementary material for: Genomic evidence for non-random endemic populations of decaying exons from mammalian genes
Source: BMC Genomics. 2009 Jul 13;10:309. doi: 10.1186/1471-2164-10-309 (PMC2718932; doi:10.1186/1471-2164-10-309)
Supplement: Additional file 4 — Examination of buffer dependence. Number of ΨEs detected, as a function of 5'/3' buffer size in the cow genome. [file 1471-2164-10-309-S4.doc]

**Supplementary Table 4: Number of ΨEs detected, as a function of 5’/3’ buffer size in the cow genome**

| **buffer size (in nucleotides)** | **number of ΨEs detected** |
| --- | --- |
| 0 | 484 |
| 1000 | 517 |
| 2000 | 533 |
| 3000 | 541 |
| 4000 | 553 |
| 5000 | 581 |
